# A study for precision diagnosing and treatment strategies in difficult-to-treat AIDS cases and HIV-infected patients with highly fatal or highly disabling opportunistic infections

**DOI:** 10.1097/MD.0000000000020146

**Published:** 2020-05-15

**Authors:** Yao Li, Yan-Ming Zeng, Yan-Qiu Lu, Yuan-Yuan Qin, Yao-Kai Chen

**Affiliations:** Division of Infectious Diseases, Chongqing Public Health Medical Center, Chongqing, China.

**Keywords:** acquired immunodeficiency syndrome, opportunistic infections, toxoplasma encephalitis, treatment regimen

## Abstract

**Background:**

An increased frequency of toxoplasma encephalitis, caused by *Toxoplasma gondii*, has been reported in AIDS patients, especially in those with CD4+ T cell counts <100 cells/μL. Several guidelines recommend the combination of pyrimethamine, sulfadiazine, and leucovorin as the preferred regimen for AIDS-associated toxoplasma encephalitis. However, it is not commonly used in China due to limited access to pyrimethamine and sulfadiazine. The synergistic sulfonamides tablet formulation is a combination of trimethoprim (TMP), sulfadiazine and sulfamethoxazole (SMX), and is readily available in China. Considering its constituent components, we hypothesize that this drug may be used as a substitute for sulfadiazine and TMP-SMX. We have therefore designed the present trial, and propose to investigate the efficacy and safety of synergistic sulfonamides combined with clindamycin for the treatment of toxoplasma encephalitis.

**Methods/Design:**

This study will be an open-labeled, multi-center, prospective, randomized, and controlled trial. A total of 200 patients will be randomized into TMP-SMX plus azithromycin group, and synergistic sulfonamides plus clindamycin group at a ratio of 1:1. All participants will be invited to participate in a 48-week follow-up schedule once enrolled. The primary outcomes will be clinical response rate and all-cause mortality at 12 weeks. The secondary outcomes will be clinical response rate and all-cause mortality at 48 weeks, and adverse events at each visit during the follow-up period.

**Discussion:**

We hope that the results of this study will be able to provide reliable evidence for the efficacy and safety of synergistic sulfonamides for its use in AIDS patients with toxoplasma encephalitis.

**Trial registration:**

This study was registered as one of 12 clinical trials under the name of a general project at chictr.gov on February 1, 2019, and the registration number of the general project is ChiCTR1900021195. This study is still recruiting now, and the first patient was screened on March 22, 2019.

## 1 Introduction

*Toxoplasma gondii* is an obligate intracellular protozoan parasite, and can infect a wide variety of warm-blooded animals, including human beings, and is responsible for the development of toxoplasmosis in humans.^[1,2]^ Over one billion people in the world are estimated to be infected with *Toxoplasma gondii*,^[3]^ and the pooled worldwide prevalence of *Toxoplasma gondii* infection is 35.8%.^[4]^

*Toxoplasma gondii* infection in healthy humans is usually asymptomatic,^[5]^ whereas AIDS patients infected by the parasite often present with toxoplasmic (or toxoplasma) encephalitis. An increased frequency of toxoplasma encephalitis has been reported in patients with AIDS, especially in those with CD4+ T cell counts of <100 cells/μL.^[6]^ The prevalence of co-infection with *Toxoplasma gondii* and HIV ranges from 25.1% to 60.7% in different countries,^[4]^ clearly indicating that co-infection with *Toxoplasma gondii* and HIV continues to be a substantial global public health concern.^[7]^

Several guidelines^[8,9]^ recommend the combination of pyrimethamine, sulfadiazine, and leucovorin for the treatment of AIDS-associated toxoplasma encephalitis. However, limited availability of pyrimethamine and sulfadiazine in China prevents the utilization of this regimen locally. Instead, healthcare providers in China usually prescribe cotrimoxazole (TMP-SMX), together with azithromycin or clindamycin as alternative therapeutic regimens in clinical practice. The synergistic sulfonamides tablet is a Chinese drug formulation that contains a combination of trimethoprim (TMP), sulfadiazine and sulfamethoxazole (SMX), and has been used for the treatment of respiratory tract infections, urinary tract infections, gastro-intestinal infections, and for acute otitis media since it was first marketed in China in 1976. We hypothesize that this drug may be used as a substitute for the recommended preferred regimens due to its compositional constituent components, sulfadiazine, TMP, and SMX. We thus designed the present trial, which proposes to investigate the efficacy and safety of synergistic sulfonamides tablet use combined with clindamycin, as a substitute for conventional recommended therapeutics in patients with AIDS-associated toxoplasma encephalitis.

## 2 Methods/design

### 2.1 Research objective

This study aims to investigate the efficacy and safety of synergistic sulfonamides, in combination with clindamycin, in patients with AIDS-associated toxoplasma encephalitis, by comparing outcomes with that of TMP-SMX plus azithromycin, a guidelines-recommended alternative anti-toxoplasma regimen.

### 2.2 Study design

**Figure 1:**
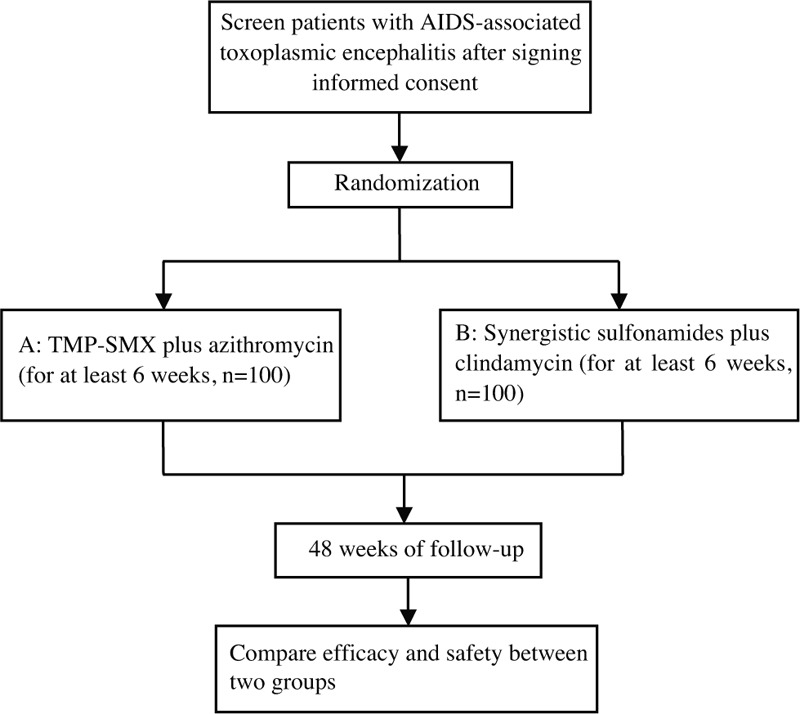
Flow chart of enrolment, intervention and follow-up.

This is an open-labeled, multi-center, prospective, randomized, and controlled trial. Two hundred patients will be recruited from the following 17 hospitals: Chongqing Public Health Medical Center, Beijing Youan Hospital of Capital Medical University, Harbin Medical University, the Second People's Hospital of Tianjin, the First Hospital of Changsha, the Eighth People's Hospital of Guangzhou, Liuzhou General Hospital, the Third People's Hospital of Guilin, the Third People's Hospital of Shenzhen, Guiyang Public Health Clinical Center, Public Health Clinical Center of Chengdu, the Third People's Hospital of Kunming, Yunnan AIDS Care Center, the Fourth People's Hospital of Nanning, Guangxi Longtan Hospital, the First Affiliated Hospital of Zhejiang University, and Xixi Hospital of Hangzhou. This protocol was written in accordance with the Standard Protocol Items Recommendations for Interventional Trials (SPIRIT) statement.^[10]^ Enrolment, intervention, and assessment processes and procedures are shown in Figure 1. All eligible patients will participate in the study voluntarily after signing informed consent, and will be invited to participate in 48-week of follow-up after starting anti-toxoplasma treatment.

Study visits will be scheduled at week 1, week 2, week 4, week 6, week 8, week 12, week 24, week 36, and week 48. All screening items will be checked at each visit, including toxoplasma antibodies, hematological analysis, urinalysis, clinical chemistry studies, lymphocyte subset, quantitative plasma HIV-1 RNA viral load, and head CT or head MRI, during the follow-up period are listed in Table 1.

**Table 1:**
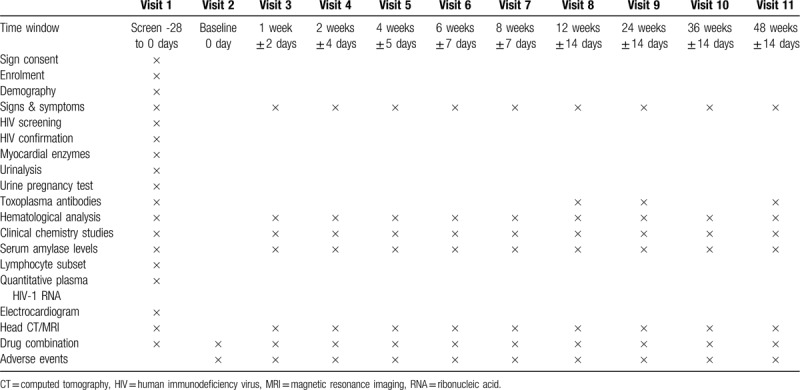
Screening items at each visit during the follow-up period.

## 3 Participants

### 3.1 Diagnostic criteria

The diagnostic criteria for AIDS-associated *toxoplasma encephalitis* in this study are consistent with the 2018 Chinese Guidelines for Diagnosis and Treatment of HIV/AIDS^[8]^ and the DHHS Guidelines for the Prevention and Treatment of Opportunistic Infections in HIV-Infected Adults and Adolescents.^[9]^ All of the following criteria have to be satisfied to make a definitive diagnosis of toxoplasma encephalitis in an AIDS patient:

(1) Clinical manifestations such as headache, focal neurological deficit, fever, mental confusion, seizures, psychomotor, or behavioral changes;
(2) CT or MRI scans show cerebral ring-enhancing lesions, especially in the basal ganglia;
(3) Positive anti-*Toxoplasma* IgG or IgM antibody test, *Toxoplasma* antigen test, *Toxoplasma* DNA detection by polymerase chain reaction (PCR), or *Toxoplasma gondii* staining;
(4) Effectiveness of anti-*Toxoplasma* treatment.

### 3.2 Inclusion criteria

Patients will be included in our study if they meet the following criteria:

(1) Aged 18 years or older;
(2) Confirmed diagnosis of AIDS-associated *toxoplasma encephalitis*;
(3) Willing to sign the informed consent.

### 3.3 Exclusion criteria

Patients will be excluded from our study if they meet the following criteria:

(1) Allergic or intolerant to therapeutic medications;
(2) Hemoglobin (Hb) <60 g/L, white blood cell count (WBC) <1.0 × 10^9/L, neutrophil count (N) <0.5 × 10^9/L, platelet count (PLT) <50 × 10^9/L, blood amylase (AMS) >2 × UNL, serum creatinine (Scr) >1.5 × UNL, aspartate aminotransferase (AST) /alanine aminotransferase (ALT) /alkaline phosphatase (ALP) >5 times of UNL, total bilirubin (TB) >2 × UNL, serum creatine phosphokinase (CK) >2 × UNL;
(3) Unstable severe concomitant diseases, opportunistic infections other than toxoplasmic encephalitis, or mental illnesses that may affect efficacy assessment or compliance;
(4) Pregnant or breastfeeding women;
(5) Intravenous recreational drug use;
(6) Non-Chinese nationality.

### 3.4 Randomization

A specific random number sequence will be generated by Medical Research Platform (http://www.51yyt.org/FrontPage/login.aspx?Inviter=) for each patient with consent, and will be used to randomly assign patients into the TMP-SMX plus azithromycin group, or the synergistic sulfonamides plus clindamycin group at a 1:1 ratio.

### 3.5 Data collection and quality assurance

All data will be documented on case report forms (CRFs) and immediately recorded in the database at Medical Research Platform independently. Missing values will be checked to ensure data completeness. Significantly abnormal data or data that are outside the clinically acceptable range (exceeding 20% of the normal value) must be explained by the attending physician. Drop-outs and adverse events will also be appropriately recorded in time.

### 3.6 Intervention

Two interventions are listed below:

Regimen A: TMP-SMX (oral dose of 1.44 g, every 8 hours) plus azithromycin (intravenous dose of 0.5 g, once a day), for at least 6 weeks; Regimen B: Synergistic sulfonamides (oral dose of 1.44 g, every 8 hours) plus clindamycin (intravenous dose of 0.6 g, every 6 hours), for at least 6 weeks.

Participants will be prescribed either Regimen A or Regimen B as their specific anti-*Toxoplasma* therapeutic modality. Participants will also receive anti-convulsants and osmotherapeutic agents during hospitalization, depending on their illness conditions, and will receive standard antiretroviral therapy (ART) at local guideline-recommended initiation temporal points subsequent to starting anti-*Toxoplasma* treatment.

The prescribed treatment regimen will be altered or changed if patients have completed the prescribed course of treatment, but have not achieved clinical or radiological improvement of their condition. After successful anti-*Toxoplasma* treatment, all patients will take TMP-SMX (oral dose of 1.44 g, once daily) for *Toxoplasma* prophylaxis for at least 3 months, or until their CD4+ T cell count cell counts reach 200 cells/μl.

### 3.7 Study outcomes

The primary study outcomes are clinical response rate and all-cause mortality at 12 weeks. The secondary outcomes are clinical response rate and all-cause mortality at 48 weeks, and adverse events at each visit. Clinical response is defined as one of the following:

(1) symptoms associated with toxoplasma encephalitis resolve after treatment;

(2) no appearance of new clinical or radiological evidence of toxoplasma encephalitis, or previously existing clinical or radiological evidence have not deteriorated, in two consecutive head MRI or CT scans.

### 3.8 Sample size

The sample size will be 100 subjects per treatment group, in order to provide at least 80% power, and an overall two-side alpha level of 0.05. The lost-to-follow-up rate will be assumed to be 15% in our study.

### 3.9 Data analysis

The primary and secondary endpoint analysis will be conducted using the Intent-to-Treat Exposed (ITT-E) and the per-protocol (PP) analysis set. The ITT-E analysis set consists of all randomized patients, whether they are in full compliance with the study protocol or not, and the PP analysis set excludes patients who do not follow the study protocol. If any data are not recorded due to drop-outs, the last observation carried forward (LOCF) method will be used to ensure the accuracy of experimental conclusions. Primary and secondary outcomes will be compared between the two groups using time-to-event methods with Cox proportional-hazards models. Categorical variables will be reported as number and percentage, and compared using Fisher exact test, and continuous variables will be reported as mean with standard deviation (SD), or median with interquartile range (IQR), and compared via the analysis of variance (ANOVA), and the Kruskal-Wallis non-parametric test.^[11]^ A *P* value of <.05 will be deemed to confer statistical significance.

### 3.10 Ethics and dissemination

The study was approved by The Ethics Committee of Chongqing Public Health Medical Center (No. 2019-003-02-KY). We will share the results through a published medical journal article and conference presentation subsequent to study completion.

## 4 Discussion

The synergistic sulfonamides tablet is an old Chinese drug formulation comprising a combination of three drugs, TMP, sulfadiazine and SMX, and was first marketed in China in 1976. The combination tablet was formulated out of consideration for saving of raw materials in order to reduce production costs in China, because the amount of SMX in each synergistic sulfonamides tablet amounts to only half of the quantity in TMP-SMX tablets.^[12]^ Despite the reduced quantity of SMX, synergistic sulfonamides have been proven to have same antibacterial effect in vitro and in vivo as TMP-SMX, as a result of consolidation of a second sulfonylurea drug, sulfadiazine, into the pharmacological formulation.^[12]^ In modern times, synergistic sulfonamides tablet is not as commonly prescribed in China, thanks to the more abundant supply of more efficacious and newer antibiotics being available on the local pharmaceutical market. Since the availability of sulfadiazine as a single drug formulation is still limited in China, and the number of patients in China with AIDS-associated toxoplasma encephalitis remains substantial, we therefore deliberated over the use of synergistic sulfonamides tablet as a substitute for sulfadiazine in the recommended therapeutic regimens for managing this patient population. Robust and reliable clinical evidence for its efficacy and safety for use in toxoplasma encephalitis is required in order to confidently advocate its widespread utilization for this purpose.

In the present study, we will compare the efficacy and safety of 2 intervention regimens: TMP-SMX plus azithromycin, and synergistic sulfonamides plus clindamycin. We would have been able to better substantiate the efficacy and safety of synergistic sulfonamides if the preferred regimen containing sulfadiazine could have been used as a control regimen. However, by comparing synergistic sulfonamides plus clindamycin with TMP-SMX plus azithromycin, with the latter regimen being a guideline-recommended alternative regimen, we will still be able to observe robust comparative investigational data for or against the use of synergistic sulfonamides as a viable alternative to current guideline-recommended therapeutic interventions.

There will be a few challenges during course of implementation of this study. The number of newly diagnosed AIDS-associated toxoplasma encephalitis cases is decreasing as a result of widespread ART coverage in China. Consequently, we may not achieve our target cohort population in the proposed study timeframe. In addition, the 48-week follow-up period may be challenging for some patients, and the drop-out rate may be higher than we have anticipated. In order to overcome the challenges that we may face, and to ensure high-quality implementation of the study, we will use the following strategies in response to these challenges:

(1) we will recruit participants from 17 large designated hospitals for HIV care;
(2) we will educate patients with regards to the aims and outcomes of our study as comprehensively as possible to enhance their compliance with our study protocol; and
(3) we will provide a superior level of consulting service excellence to participants during the study period, which will extend to the period after study completion.

## 5 Trial status

This trial is currently in the recruitment phase. Patient recruitment began in March 2019 and is expected to be completed in May 2020.

## Author contributions

Yao Li and Yan-Ming Zeng drafted the original manuscript. Yan-Qiu Lu completed the study registration, and edited the manuscript. Yuanyuan Qin conducted the literature search and obtained ethics approval. Yaokai Chen conceived and designed the study, revised the final manuscript, and acquired funding.
